# Survey on Dental Students’ Knowledge of Infection Prevention and Control Rules

**DOI:** 10.3390/dj14030153

**Published:** 2026-03-09

**Authors:** Velina Stoeva, Tsonka Miteva-Katrandzhieva, Elizabeth Shamsee

**Affiliations:** 1 Department of Epidemiology and Disaster Medicine, Medical University, Bulevard Vasil Aprilov 15A, 4002 Plovdiv, Bulgaria; velina.stoeva@mu-plovdiv.bg; 2 Department of Social Medicine and Public Health, Medical University, Bulevard Vasil Aprilov 15A, 4002 Plovdiv, Bulgaria; 3 Dental Faculty, Medical University Plovdiv, Vasil Aprilov 15A, 4002 Plovdiv, Bulgaria; elizabethshamsee@hotmail.com

**Keywords:** infection prevention, dental education, disinfection, dental students, cross-contamination, prosthetic impressions, infection control, epidemiology

## Abstract

**Background/Objectives:** This study evaluates the level of knowledge among dental students regarding infection prevention and control measures in dental practice. **Methods:** A total of 225 students from the Medical University of Plovdiv participated in an anonymous survey between November 2024 and March 2025. The survey assessed knowledge about epidemiological factors, disinfection protocols, and procedures for infection prevention and control. **Results:** The survey results revealed that while most students demonstrated a high level of knowledge of the importance of protective equipment and impression disinfection, a few students reported incorrect or inconsistent practices. Gender differences were observed in adherence to disinfection procedures. **Conclusions:** The findings highlight the need for enhanced training in infection control within dental curricula to ensure safe clinical practice and minimize cross-infection risks.

## 1. Introduction

Epidemiological factors for the transmission of infections are elements of the external environment where microorganisms can survive for a certain period, remaining viable until they pass to another new susceptible organism. It has long been established that numerous microorganisms pose a threat to patients, dentists, auxiliary personnel and, of course, to dental students during the provision of dental care involving patient contact. A confirmed case of patient-to-patient transmission of hepatitis B virus (HBV) in oral surgery was reported. Infection transmission was between patients and was associated with infection control violations. Genetic analysis of the virus shows an identical strain and confirms that failure to follow standard precautions can lead to serious blood-borne infections in dental practice [1]. Another study [2] reports an outbreak of HBV, with five cases linked to a single clinic in the United States. Improper sterilization, reuse of supplies, and poor control of injection practices have been cited as the main causes. If infection control standards are not followed, the risk of transmission of infections is real.

This underlies the need for precise and clear rules for safe work, which are essential for the prevention and spread of dental care-associated infections. In dental infection control, standard precautions should always be applied when in contact with all body fluids (blood, saliva, secretions, regardless of whether they contain blood), as well as with nonintact skin or mucous membranes. The risk of infection transmission in dental practice is associated with the presence of infectious sources, which includes patients or members of the dental team who are sick or infected. However, in all cases, it must be assumed that each participant in the treatment is potentially infected, even if no symptoms are apparent at the time of examination. This requires strict adherence to the procedures for infection prevention and control, both before and during work in the dental practice [3,4,5,6,7].

A significant part of dental services in Bulgaria is provided by dental students who are in their fourth, fifth and sixth year of their six-year education. This raises the logical question to what extent they are familiar with the procedures for infection prevention and control and whether they comply with them during the provision of dental care. During the third year, students have preclinical practices in which they work on plastic phantom models. This is the first time they acquire knowledge, skills, and create habits related to their work with future real patients. Fourth-year dental students study about infectious disease epidemiology and they are introduced to details to the procedures for infection prevention and control they must follow in the dental office. Lack of knowledge or non-compliance will lead to a violation of the gold-standard infection prevention and control procedures [7]. Dental students are also observed and encouraged by their Assistant Professors during their clinical practice with patients to adhere strictly to these rules. Only the strict implementation of such protocols in daily dental practice can ensure a safe working environment for students, dental assistants and patients.

The aim of this study was to assess the level of knowledge among dental students regarding the main factors for the transmission of infections in dental practice, as well as their knowledge of the rules and methods of disinfection, which are key to reducing the spread of infectious diseases in dental practices and dental laboratories. The working hypothesis was the presence of a high level of knowledge about ways to prevent the spread of infections, as well as strict implementation of disinfection protocols. This study also seeks to identify potential gaps in knowledge and to develop recommendations for student training and practice that will strengthen infection prevention and control, ultimately contributing to improved public health.

## 2. Materials and Methods

An anonymous survey was conducted using the direct group survey method. The survey questions assessed the level of knowledge of procedures for infection prevention and control, the correct use of personal protective equipment, hand disinfection—when and for how long—proper decontamination of the workplace, proper disinfection of the impressions taken from the prosthetic field, as well as the finished dental appliance. The respondents, third-year students (still working only on phantom models) and fourth- and fifth-year students (performing clinical work with patients) completed a questionnaire consisting of 17 questions, designed specifically for the purposes of this study.

To determine a representative sample, several standard assumptions were applied: confidence level: 95%, margin of error: ±5%, expected proportion (p): 50%.

The minimum required sample size to meet the representativeness requirement was approximately 216 students. Our sample consisted of 225 respondents.

Students were given an informed consent form in advance to participating in this study. The purpose and contribution of the obtained information were explained to them. An assurance that the information from the study would be used only for scientific purposes and only in a summarized form was underlined. The survey was administered in the presence of the authors who are academic lecturers, thus providing the opportunity for clarifying questions from students. This method ensured both anonymity and an accurate assessment of the students’ actual level of knowledge.

Social desirability bias was reduced by the study design. The highest level of honest answers is available when implementing a direct group survey as it ensures anonymity and confidentiality. Anonymous surveys substantially reduce socially desirable responding.

The study period was November 2024–March 2025 and was conducted in the territory of the Medical University of Plovdiv. Response rate was 45.73%.

The systematization, processing and analysis of the primary data were implemented with the Statistical Package of the Social Science software IBM SPSS Statistics v. 19. For all tests, a significance level of α = 0.05 was adopted. The analysis, conclusions and recommendations from this study are derived after the summary presentation of the empirical results in tabular form or illustrated with their corresponding graphic images. The graphic analysis was performed in the MS Office 365 environment using Excel.

The following statistical methods were used to objectify the results of the analyses:

1. Descriptive analysis to describe the structure of the studied variables
  • Descriptive statistics for quantitative variables—quantitative description of the main properties and characteristics of the dataset; summarization and evaluation of the main statistical parameters. Normally distributed data are presented as the mean value (mean) ± standard deviation (SD).
  • Descriptive statistics for qualitative variables—absolute and relative frequencies. Presented, respectively, as ordinary numerical values (n) and as a relative share (%).
2. Testing statistical hypotheses
  • Non-parametric analysis:

χ^2^—to check the presence of associations.

The comparisons made in this study are based on age, gender and nationality (data obtained from Bulgarian and foreign students were compared).

3. Graphical analysis

The presentation of the results of the analyses was carried out through frequency tables (multivariate frequency distribution tables), containing the following:

 • Absolute frequencies—the number of units in a separate group.
 • Measures of central tendency and measures of dispersion.
 • Relative frequencies—the number of units in a separate group relative to the total number of units in the population.
 • *p*-values.

## 3. Results

This study included a total of 225 students, of which 70.67% (n = 159) were Bulgarian students and 29.33% (n = 66) were foreign students, with 43.56% (n = 98) men and 56.44% (n = 127) women. No one identified as another gender. The distribution by year of study is as follows: 1.33% (n = 3) were in their third year, 98.22% (n = 221) were in their fourth year and 0.44% (n = 1) were in their fifth year (Table 1).

Procedures for infection prevention and control in a dental practice are a set of measures designed to prevent the transmission of infectious diseases between patients, dental staff, and the clinical environment. It includes patient screening; strict hand hygiene; use of personal protective equipment (PPE) such as masks, gloves, and face shields; and proper sterilization and disinfection of instruments and surfaces. The protocol also covers safe waste disposal, adequate ventilation, and procedures for managing suspected or confirmed infectious cases. Together, these measures reduce the risk of cross-infection and ensure a safe clinical setting. The analysis of the results shows that a proportion of the students underestimate the use of protective gloves as a means of protection against HAI (healthcare-associated infections)—92.44% (n = 208) work with gloves, 4.89% (n = 11) without, and for 2.67% (n = 6), it does not matter whether they carry out diagnostic or therapeutic activities with or without gloves.

**Figure 1:**
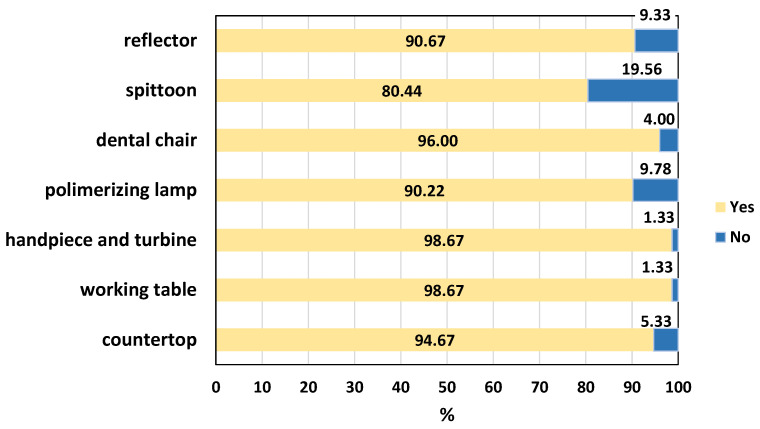
Disinfection areas.

Equally important in infection control is the decontamination of surfaces following the provision of dental care. The analysis shows that the same students who disregarded the rules for preventing the spread of infection in dental practice cleaned the surfaces without gloves, thereby exposing themselves and others to a significant risk of infection with blood-borne pathogens (Figure 1). As can be seen from the figure, according to 90.67% (n = 204), the reflector should be disinfected, but 9.33% (n = 21) do not indicate it as necessary. Regarding the spittoon, we again do not achieve optimal results, with 80.44% (n = 181) disinfecting it but 19.56% (n = 44) not doing so. The remaining positive answers are as follows: the dental chair should be disinfected according to 96.00% (n = 216), the photopolymerization lamp—according to 90.22% (n = 203), the turbines and handpieces, as well as the work table are subject to disinfection according to almost all respondents, 98.67% (n = 222), the operating buttons according to 86.67% (n = 195).

Only the complete disinfection of all these objects would lead to a reliable interruption of cross-contamination in the dental office. No statistically significant difference was found between the two genders and between Bulgarian and foreign students.

Decontamination of impressions taken from the prosthetic field should always be performed before sending them to the dental laboratory. The majority of students, 89.78% (n = 202), reported compliance with this rule. However, 8% (n = 18) of students disinfect the impressions only when visible traces of blood are present, while 2.22% (n = 5) of students believe that cleaning the impression is the responsibility of the dental technician. A statistically significant gender difference was observed: 95.28% of female students (n = 121) reported always sending decontaminated impressions to the laboratory. In contrast, among male students, the relative share of those who disinfect impressions in all cases is 82.65% (n = 81), (*p* < 0.01) (Table 2).

The sequence in which disinfection is carried out is of decisive importance for its effectiveness. According to the rules of good dental practice, impressions should first be washed with water, then dried, and only afterwards treated with disinfectant. The results of this study show that less than a third of the respondents, 31.11% (n = 70), follow this protocol. However, over half of the respondents, 56.44% (n = 127), answer that they wash the impression with water and disinfect it without drying it between these two steps. This approach is incorrect, since residual water dilutes the disinfectant, preventing it from achieving its maximum effect. Washing an impression from the prosthetic field with water alone is insufficient, but this is how 12.44% (n = 28) of the surveyed students work.

A statistically significant difference between the two genders is observed in terms of the methodology for disinfecting the taken impressions: 39.80% of men (n = 39) believe that impressions should be washed, dried and disinfected, while only 24.41% (n = 31) of women gave this answer (*p* < 0.01).

**Figure 2:**
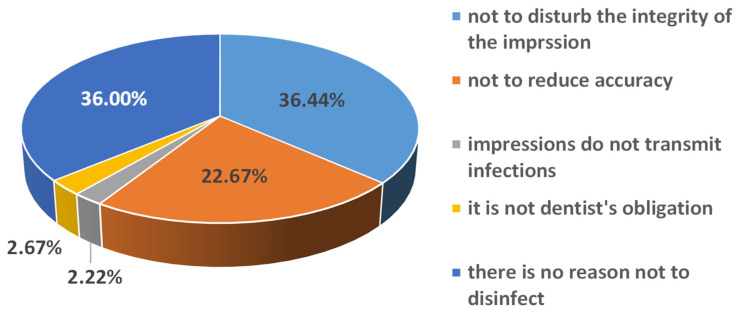
Reasons for lack of impressions disinfection.

The main reasons cited by respondents for not disinfecting impressions are related to concerns about changing the integrity or accuracy of the impression. A small proportion of respondents believe that infections cannot be transmitted through impressions or do not accept the disinfection of the impression as their responsibility (Figure 2).

**Figure 3:**
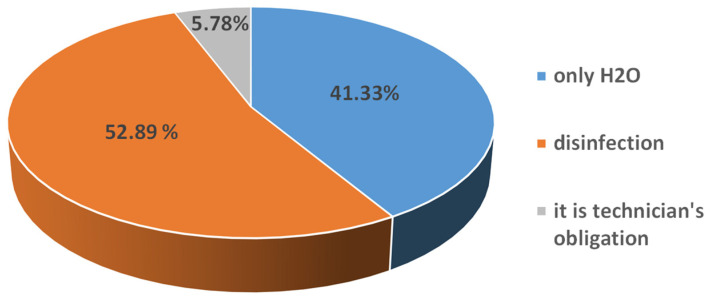
Disinfection of finished appliance.

Upon receiving the finished prosthetic appliance, the actions taken by the respondents are suboptimal: only half of the respondents reported carrying out disinfection, with a slight predominance among women, 55.12% (n = 70) (*p* < 0.01). A few students, 5.78% (n = 13), reported taking no action at all, as they consider this to be the duty of the dental technician, and 41.33% (n = 93) only wash the dentures, crowns and bridges with water, which is clearly insufficient (Figure 3).

Dental students were asked about the need to use an individual sterile kit from a dental technician in cases where the shade for the future prosthetic appliance needs to be determined: 51.56% (n = 116) answered positively. According to 29.78% (n = 67), the dental technician does not touch the patient’s mouth or face, and 16.00% (n = 36) believe that the dental technician should use their hands, using gloves, instead of an individual sterile kit. According to 2.67% (n = 6) of the students, the dental technician should use their hands without using gloves.

## 4. Discussion

According to modern protocols [7] for infection prevention and control in healthcare facilities, no material, impression or construct should leave the dental office or dental laboratory without prior disinfection.

Both the dentist and the dental technician must follow a shared disinfection protocol, and it is the responsibility of both parties to ensure that impressions and finished prosthetic appliances are disinfected. Effective control of cross-infection between the dental clinic and the laboratory is critical for safeguarding the health of the dental personnel, technicians and patients and, ultimately, impacts the individual, general public and dental health [7,8,9].

Dental personnel include dentists, dental assistants, dental technicians, hygienists and administrative staff. All may be exposed to potentially pathogenic microorganisms, such as hepatitis B virus, hepatitis C virus, *Mycobacterium tuberculosis*, staphylococci, streptococci, and other pathogens colonizing the oral cavity and upper respiratory tract [6,7,8,9,10]. The Centers for Disease Control and Prevention guidelines stress the fact that dental impressions are potential sources of cross-contamination and should always be managed in a manner that prevents exposure to dental professionals, patients, and the environment [7,8,9,10,11].

It is recommended that dental impressions be rinsed with appropriate disinfectants to remove saliva, blood, and any organic matter from them. Impressions should be rinsed under running water, avoiding dusting and splashing, to remove all visible traces of organic matter and disinfected with an appropriate impression disinfectant before being transferred to the dental laboratory, and gloves should be worn when casting. It is recommended that materials removed from the office and transferred to the laboratory be handled and stored in a designated area, and that personnel handling them wear protective clothing and disposable gloves [12].

The authors [13] studied the effect of chlorhexidine at various concentrations ranging from 0.2 to 5% and found that it provided a significant reduction in the viability of most pathogens in the biofilm but had minimal effect on *Candida albicans*. It had no effect on the dimensions of alginate dental impressions by immersion, but it did alter the surface of silicone impressions.

Commonly used are chlorhexidine, alcohol, glutaraldehyde and sodium hypochlorite, which can be applied via spraying or immersion. However, none of them are universally suitable for all methods of disinfection of impression materials. In vitro studies confirm that chlorhexidine, alcohol, glutaraldehyde and sodium hypochlorite have been shown to reduce the number of CFU/mL on the surface of impressions, supporting their role in preventing cross-contamination [14].

Disinfection of impressions and prosthetic appliances remains a key element of infection control. Our study reports a high level of knowledge about the need for disinfection of impressions taken but not optimal results in indicating the specific reasons, methods, means of disinfection, etc. The main reasons for not disinfecting are concerns that it will adversely affect the reproduction of the finished construct.

Another study compared the linear dimensional changes in synthesized tetrafunctional (dimethylsilyl) orthosilicate impressions containing polyvinylsiloxane after disinfection with sodium hypochlorite and found that prolonged immersion can negatively affect linear dimensions [15].

Ideally, disinfectants should preserve the physical and chemical properties of impression materials, including dimensional stability, surface roughness, hydrophilicity and fine detail reproduction. Factors influencing accuracy include the type of disinfectant used, the disinfection time, water content, temperature and disinfection method (immersion or spraying) [16].

New disinfection techniques such as UVC irradiation and ozone treatment are under investigation. They are sought to be effective, inexpensive, and convenient but also not adversely affect the properties of dental impressions. A study compared the effects of different methods (UVC, gaseous ozone, commercial solution, and spray) on the dimensional change, tensile strength, and hardness of silicone impressions. The results showed that both traditional (immersion and spraying) and alternative disinfection methods (UVC and ozone) do not have a significant impact on the tensile properties and dimensional stability of the silicones studied [17].

Concerns over changes in impression accuracy and material properties remain the main reason cited by respondents in our study for not performing disinfection. Nonetheless, both impressions and final prosthetic appliances must always be disinfected, with methods carefully chosen based on the impression material.

It is logical to raise questions about the main reasons leading to these results and how to improve students’ motivation for the strict implementation of disinfection protocols. The main reasons for not disinfecting are concerns that it will adversely affect the dental appliance. Improving the implementation of disinfection protocols is possible through providing additional information to dental students during practical exercises and lecture courses in the Department of Orthopedics and Materials Science and the Department of Epidemiology, regarding the lack of significant changes in the accuracy of impression materials and impression quality after disinfection. It is possible to propose to the manufacturers of disinfectants used in dentistry to provide such information in the brochures and on the labels of their products.

The findings of this study are not unique to Bulgaria; similar results have been reported by other author teams. A detailed review of the available literature confirms that inadequate adherence to infection control in impression disinfection is a global issue, warranting further discussion to identify poor practices and propose solutions. An interesting study by a team of authors [18] compared the attitudes and behaviors of fourth-year dental students regarding infection control regulations in 1995 and 2005, covering 592 students, finding alarming results. No improvement was observed in the use of rubber gloves (*p* = 0.316), face masks (*p* = 0.572) or aprons (*p* = 0.862) between 1995 and 2005. A lower frequency of the use of safety glasses was observed in 2005 (*p* < 0.001). No student used personal protective equipment correctly. The results are similar to our reported responses on glove use—92.44% (n = 208) work with gloves, 4.89% (n = 11) without, and for 2.67% (n = 6), this does not matter. The data indicate suboptimal compliance with infection prevention measures in dental practice by the students we surveyed.

A study in Jordan involving 190 dental practitioners focused on recommended infection control guidelines. The following results were found: The majority of respondents washed their hands before (66.3%) and after (83.2%) treatment. Approximately 87.9% of them wore gloves, and 78.9% wore masks while performing dental procedures. In the study, dental assistants showed low adherence to infection control guidelines compared to dentists [19].

Our results appear more favorable, though this may also be because our respondents were students working under the supervision of an Assistant Professor during their clinical practice.

Another study assessed the implementation of basic routine measures to prevent cross-infection in a dental clinic at the University College Hospital, Ibadan. The sample consisted of 77 staff in different clinics who completed closed-ended questionnaires. The questionnaires, as in our study, were aimed at compliance with work protocols. The results highlighted poor compliance with the hospital’s hepatitis B vaccination program by employees, especially dental surgeons and students [20].

Hepatitis B and C are among the most common blood-borne pathogens in dental practice, given the specifics of dental work. A reliable basis for preventing their cross-transmission between patients and the dental team is to raise knowledge of the prevention and control of blood-borne infections among dental personnel and infections associated with dental care in general [21,22,23,24].

A study [25] assessing students’ knowledge of cross-infection prevention in dental practice, including proper impression handling, found that infection control knowledge was not excellent and highlighted the need for enhanced training on disinfection issues. This conclusion is also reached by us based on the results obtained.

The reasons for the high risk in dental practice are many, but among the most important are the low infectious dose, work with sharp and cutting instruments, and a lack of knowledge about the infectious status of the patient [26].

It is important to note that in Bulgaria, vaccination against this infection has been part of the mandatory immunization schedule for our country since 1992, but those born before that, unless they are vaccinated of their own free will, are not protected [27].

## 5. Conclusions

Infection prevention remains a topic of enduring importance. Dental students must possess thorough knowledge of the epidemiology and protocols for infection control and be able to apply this knowledge in clinical practice. Disinfection methods must be carefully tailored to the properties of impression materials to avoid compromising their accuracy. Both the choice of disinfectant and the protocol of application are critical to ensuring reliable impressions while minimizing cross-infection risk.

While most respondents in this study demonstrated adherence to infection control measures, gaps were evident. Targeted educational programs and enhanced training strategies should, therefore, be implemented to improve compliance with infection control guidelines among future dental practitioners.

## Figures and Tables

**Table 1:** Distribution of students by gender, nationality and age.

		Students		Total
Gender	Age	Bulgarians	Foreigners	
**Men**	up to 24 years	61 (84.7%)	23 (88.5%)	84 (85.7%)
	over 24 years	11 (15.3%)	3 (11.5%)	14 (14.3%)
	Total	72 (100%)	26 (100%)	98 (100%)
**Women**	up to 24 years	77 (88.5%)	36 (90%)	113 (89%)
	over 24 years	10 (11.5%)	4 (10%)	14 (11%)
	Total	87 (100%)	40 (100%)	127 (100%)
**Total**	up to 24 years	138 (86.8%)	59 (89.4%)	197 (87.6%)
	over 24 years	21 (13.2%)	7 (10.6%)	28 (12.4%)
	Total	159 (100%)	66 (100%)	225 (100%)

**Table 2:** Impression disinfection associated with gender.

			Impression Is Disinfected			Total
			Yes	In Case of Blood Presence	No, It Is Laboratory Technician’s Obligation	
Gender	Men	Count	81	13	4	98
		% within impression	40.1%	72.2%	80.0%	43.6%
	Women	Count	121	5	1	127
		% within impression	59.9%	27.8%	20.0%	56.4%
Total		Count	202	18	5	225
		% within impression	100.0%	100.0%	100.0%	100.0%

χ^2^ = 9.7, (*p* < 0.01).

## Data Availability

The original contributions presented in this study are included in the article. Further inquiries can be directed to the corresponding author.
